# Assessment of Geographic and Host-Associated Population Variations of the Carob Moth, *Ectomyelois ceratoniae*, on Pomegranate, Fig, Pistachio and Walnut, Using AFLP Markers

**DOI:** 10.1673/031.008.0601

**Published:** 2008-01-25

**Authors:** Fariba Mozaffarian, Mohsen Mardi, Alimorad Sarafrazi, Gadir Nouri Ganbalani

**Affiliations:** ^1^ Insect Taxonomy Research Department, Iranian Research Institute of Plant Protection, Tehran, 19395-1454, Iran; ^2^ Department of Genomics, Agricultural Biotechnology Research Institute, Karaj, Iran; ^3^ Insect Taxonomy Research Department, Plant Pests and Diseases Research Institute, Tehran, 19395-1454, Iran; ^4^ Faculty of Agriculture, Moghadas-e Ardebili University, Ardebil, Iran

**Keywords:** *Punica granatum*, *Ficus carica*, *Pistacia vera*, *Juglans regia*

## Abstract

The carob moth, *Ectomyelois ceratoniae* (Zeller 1839) (Lepidoptera: Pyralidae) is the most important pest of pomegranate, *Punica granatum* L. (Myrtales: Ponicaceae), in Iran. In this study, 6 amplified fragment length polymorphism primer combinations were used to survey the genetic structure of the geographic and putative host-associated populations of this pest in Iran. An AMOVA was performed on test populations. Pairwise differences, Mantel test, multidimensional analysis, cluster analysis and migration rate were calculated for 5 geographic populations of *E. ceratoniae* sharing the same host, pomegranate. In another part of the study, 3 comparisons were performed on pairwise populations that were collected on different hosts (pomegranate, fig, pistachio and walnut) in same geographic regions. The results showed high within population variation (85.51% of total variation), however geographic populations differed significantly. The Mantel test did not show correlations between genetic and geographic distances. The probable factors that affect genetic distances are discussed. Multidimensional scaling analysis, migration rate and cluster analysis on geographic populations showed that the Arsanjan population was the most different from the others while the Saveh population was more similar to the Sabzevar population. The comparisons didn't show any host fidelity in test populations. It seems that the ability of *E. ceratoniae* to broaden its host range with no fidelity to hosts can decrease the efficiency of common control methods that are used on pomegranate. The results of this study suggest that in spite of the effects of geographic barriers, high within-population genetic variation, migration rate and gene flow can provide the opportunity for emerging new phenotypes or behaviors in pest populations, such as broadening host range, changing egg lying places, or changing over-wintering sites to adapt to difficult conditions such as those caused by intensive control methods.

## Introduction

Improper identification or inability to recognize distinct populations can have drastic and costly consequences for pest management (Menken and Ulenberg 1987). It is believed that intra-specific variations that lead to the emergence of biotypes, hosts or pheromone races may cause different reactions to control methods (Menken and Raijman 1996). Allopatric and sympatric speciation have been described during the divergence of populations (Mayr and Aschlock 1991; Kirkpatrick and Ravigne 2002). In allopatric speciation, geographic barriers among distinct populations may prevent gene flow (Mayr and Aschlock 1991). Given the lack of gene flow separate processes of variation in different geographic areas may result in divergence in ethological (Mitani et al. 1999), physiological (Tauber et al. 1986; Ruberson et al. 2001) and morphological (Dujardin et al. 1999; Rodrigues and Moreira 2002; Jennions and Kelly 2001) characters in any isolated population. There may be other factors, such as human transportation, which cause more gene flow among allopatric populations and decrease the efficiency of geographic barriers (Failloux et al. 1997; Julvez et al. 1990; Julvez and Mouchet 1994; Chen et al. 2004; Goodisman et al. 2001). In sympatric speciation variation may be the result of adaptation to different habitats such as hosts in some localities that is more possible in the presence of assortative mating which decreases gene flow among such populations (Kirkpatrick and Ravigne 2002). Nason et al. 2002 believed that careful studies of apparently generalist phytophagous insects often revealed that they instead represent complexes of genetically differentiated host races or cryptic species. If host species constitute different selective regimes to herbivorous insects, genetic differentiation and host-associated local adaptation may occur (Ruiz-Montoya et al. 2003), which is definitely important in pest management programs. Host fidelity in true fruit flies of the genus *Rhagoletis* L. (Diptera: Tephritidae) (Feder et al. 1990 and 1993) and also the palaearctric genus *Yponomeuta* Latreille (Lepidoptera: Yponomeutidae) (Menken et al. 1992) are the most important examples of sympatric speciation (Menken and Raijmann 1996).

Among different data sets, genetic data are basic to the design and successful application of any pest management strategy (Sluss and Graham 1979). Estimating genetic variation and genetic structure of natural populations relies on genotyping individual specimens (Yan et al. 1999). The analysis of genetic variation using DNA markers has become an important approach for assessing the population genetics of a variety of insect species (Reineke et al. 1998). To analyze the genetic data, several techniques can be used. The amplified fragment length polymorphism (AFLP) technique is a powerful method for population genetic studies that was described by Vos et al. 1995. This method has been applied in many aspects of insect population studies such as distinguishing different geographic populations of gypsy moth, *Lymantria dispar* L. (Reineke 1998), host-associated strains of fall army worm, *Spodoptera frugiperda* (McMichael and Powell 1999) and indicating host-associated lineages of the snakeweed grasshopper, *Hesperotettix viridis* (Sword et al. 2005)

Pomegranate, *Punica granatum* L. (Myrtales: Ponicaceae), is the main host of the carob moth, *Ectomyelois ceratoniae* (Zeller 1839) (Lepidoptera: Pyralidae) in Iran. Larvae feed on inner parts of the fruit and highly reduce its quality indices. Several other host plants of *E. ceratoniae*, such as citrus, date and almond have been recorded (Morton 1987; Alrubeai 1987; Warner et al. 1990; Van den Berg 1995; Tous and Ferguson 1996; Mesbah et al. 1998; Bouka et al. 2000). The activity of *E. ceratoniae* on fig (Shakeri 1993) and pistachio (Mehrnejad 2002) has been described in Iran. The most recommended control method in Iran is collecting and destroying infected pomegranates that eliminate over-wintering sites at the end of growth season (Behdad 1991). This control method has also been used for macadamia (Van den Berg 1995). Biological control was used by Nasrollahi et al. 1998. Two other methods, staffing the pomegranate fruit neck (Mirkarimi 1966) and removing its flags (Shakeri 2004) were suggested to eliminate the sites that the moth uses to lay eggs. These methods make these places inconvenient for laying eggs. In spite of the important role of the genetic structure of insect populations in pest management, there is no documented information on genetic aspects of different *E. ceratoniae* populations. In this study AFLP markers were used to determine the differences and/or similarity among several geographic populations of *E. ceratoniae* in Iran on 4 hosts, pomegranate, fig, pistachio and walnut. Fidelity to hosts was examined as well.

**Table 1.**
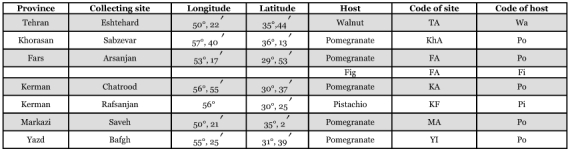
Localities, hosts and codes of collected Carob moth, *Ectomyelois ceratoniae*, populations

**Figure 1.**
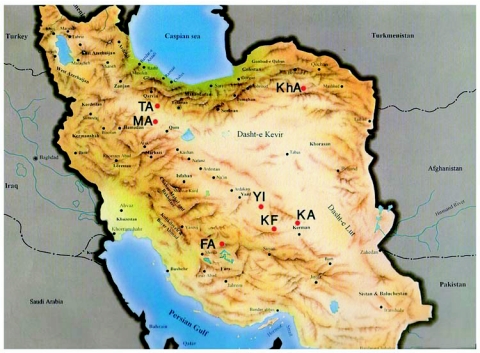
Distribution of collecting sites. See Table 1 for the codes.

## Materials and Methods

### Sample collection

A total of 180 *E.ceratoniae* larvae were collected from 7 places in six provinces of Iran on four hosts including pomegranate, *P. granatum* L., fig , *Ficus carica* L.(Urticales: Moraceae), pistachio, *Pistacia vera* L.(Sapindales: Anacardiaceae), and walnut, *Juglans regia* L. (Juglandales: Juglandaceae) (Table 1 and Figure 1). During sampling, some similar larvae belonging to other Lepidoptera could also be found inside collected fruits. These larvae were reared in their natural infected hosts to adulthood and species identification were performed on adults. Adults of *E. ceratoniae* were separated and kept at -80° C after emergence.

### DNA isolation

Total genomic DNA was isolated using a CTAB-method suggested by Reineke et al. 1998 with minor modifications. The average number of 20 individuals in any population was used for further analyses. DNA quantity and quality were measured with a UV-Photometer.

### AFLP markers

The AFLP analysis was performed using the method described by Vos et al. 1995 using the enzyme combination EcoRI and MseI. Six primer combinations (E-AAC/M-CCC, E-AAC/M-GAG, E-AAC/M-CAA, E-GTC/M-CAA, E-GTC/M-GAG and E-ACT/M-CAA) were used for selective PCR amplification. PCRs were performed on a BioRad thermocycler (BioRad Laboratories Inc., Hercules, CA, USA). Amplification reaction products were separated on a 6% denaturing polyacrylamide gel using a Sequi-Gen GT Sequencing Cell 50 cm gel apparatus (BioRad Laboratories Inc., www.bio-rad.com). The amplified fragments were detected by silver staining method as described by Bassam et al. (1991). In order to prove the lack of PCR artifacts, a subset of samples containing 10 individuals were prepared and run twice independently which resulted in similar patterns. The resulting gels were scored manually and independently by two people.

### Data analysis

An analysis of molecular variance (AMOVA) was performed to analyze genetic structure of test populations and then F_IS_ , F_ST_, and F_IT_ were calculated. Differences among 5 geographic populations (Table 1) sharing the same host (pomegranate) were detected by measuring population pairwise F_ST_ using the distance method (number of permutations: 110). This method was also used to compare two by two putative host-associated populations in same geographic regions (number of permutations: 3024). In other words, each two geographic populations on the same host (pomegranate) were compared to find any geographic effect: (5×4)/2 = 10 comparisons. The analyses were also performed for sympatric or quasi-sympatric populations on different hosts to determine any possible host effect: population on pomegranate of Arsanjan (FA, Po) versus those on fig of the same locality (FA, Fi); population on pomegranate of Chatrood (KA, Po) versus those on pistachio in the same province (KF, Pi); pomegranate of Saveh (MA, Po) versus those on walnut in Eshtehard (TA, Wa) (see Table 1 for codes). The above analyses were performed by Arlequin version 2 software (Schneider et al. 2000). The data of 5 geographic populations belonging to different geographic regions on the same host (pomegranate), were subjected to later analyses (KhA,Po; FA,Po; KA,Po; MA,Po and YI,Po; Table 1): The migration rate (M values), which is the absolute number of migrants exchanged between the two populations, were calculated (Slatkin, 1991) and the relationship between linearized genetic (F_ST_ /(1- F_ST_)) and geographic (log_10_Km) distances (Slatkin 1993) was detected by Mantel test (number of randomizations, 10,000) using Mantel version 1.15 software (Cavalcanti 2002). The resultant F_ST_ matrix was also subjected to cluster analysis by the unweighted pair group method using arithmetic means (UPGMA) using PowerMarker Ver. 3.20 (Liu 2004) to show similarity among geographic populations. A dendrogram was constructed based on 10000 bootstrap resampling. A multidimensional scaling analysis was performed on a matrix of linearized genetic distance [F_ST_ /(1- F_ST_)] using SPSS 11.5 for windows (SPSS Inc. 2002). The resulted plot showed a geometric map according to genetic distances among geographic populations.

## Results

Six AFLP selective primer combinations yielded 118 polymorphic bands. The results of AMOVA indicated high within population variation (85.51% of total variation) with fixation indices: FIS: 0.08456, *p*= 0.00000+-0.00000; F_ST_: 0.14491, *p*= 0.00000+-0.00000; F_IT_: 0.06593, *p*=0.04497+-0.00657.

**Figure 2.**
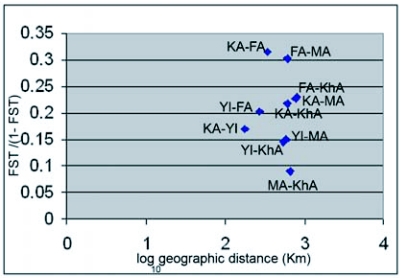
Scatter plot based on geographic and genetic distances between pairwise geographic populations of E. ceratoniae sharing the same host (Po) based on AFLP marker. See Table 1 for the codes.

Comparing 5 geographic populations that were collected on pomegranate showed significant differences (*p*=0.00000+-0.0000). Computed F_ST_ values for these comparisons were low to moderate, from 0.08194 to 0.23973 with a mean of 0.16765 (Table 2). All host pairwise comparisons within same geographic origin showed no significant F_ST_ P values (*p*= 0.99907+-0.0000). Data analysis of geographic populations showed migration rates between pairwise populations (M values) from: 1.58572 to: 5.60196 with the mean of 2.746512 (Table 3). According to the results of the Mantel test there was no correlation between genetic and geographic distances (r =-0.006, *p*=0.4938). Hence there may be some other factors that affect on decreasing or increasing genetic distance among test populations. In Figure 2, pairwise geographic populations with the lowest relationship between their geographic and genetic distances are detectable.

**Figure 3.**
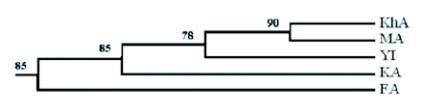
Cluster analysis of 5 tested geographic populations of E. ceratoniae using UPGMA method based on 10000 bootstrap resample. Numbers are bootstrap values. See Table 1 for the codes.

**Figure 4.**
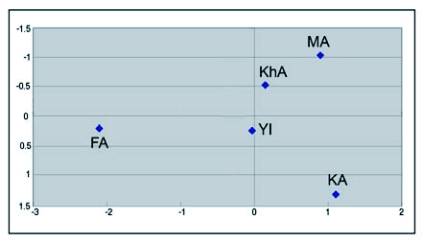
Geometric location of geographic test populations obtained from multidimensional analysis on genetic distance. See Table 1 for codes.

The migration rate among Arsanjan (FA) and other populations was rather low. It was smallest for Saveh and Sabzevar (MA- KhA) (Table 3). The results are also supported by cluster analysis (Figure 3) that showed differentiation of the Arsanjan population (FA) and similarity between the Saveh (MA) and Sabzevar (KhA) populations. Figure 4 shows the geometric map according to genetic distances resulted from multidimensional analysis. Comparing this map with the one according to geographic distances (Figure 1) reveals some changes in the relative position of populations; the Saveh (MA) population moved from northwest to northeast near Sabzevar (KhA), and the latter population is closer to the others than they are geographically. Another remarkable change in the map of genetic distance is the position of Arsanjan (FA) that is rather much farther from others than its geographic location.

**Table 2.**
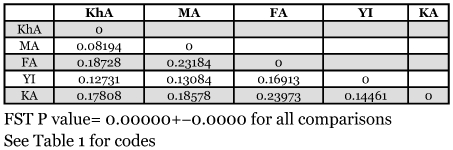
Populaiton pairwise FST of 5 tested geographic population comparisons.

**Table 3.**
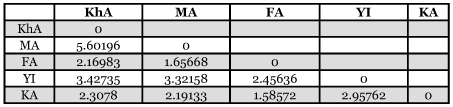
Matrix of M values (migration rates) for 5 tested geographic populations (see Table 1 for codes)

## Discussion

This analysis showed high intra population variation and small to medium significant variations among geographic populations. The significant variation has also been shown in many other insects including Lepidoptera (Coates et al. 2004; Koshio et al. 2002; Suinaga et al. 2004; Timm et al. 2006). Since test geographic populations were all associated with the same host (pomegranate), population differences may have been mainly the result of geographic barriers that are defined as any terrain that prevents gene flow between populations (Mayr and Ashlock 1991). Sometimes distance alone can function as a barrier to genetic exchange among populations (Ruggiero et al. 2004). The Mantel test did not show correlations between geographic and genetic distances in test populations. Figure 2 shows that distances associated with the two Arsanjan (FA) populations correspond to high genetic distances in spite of rather short geographic distances. It can be concluded that geographic distances do not completely explain genetic distances associated with FA and there should be some other factors that isolate and decrease gene flow of this population to the others.

The differentiation of FA is also confirmed by calculating migration rate (Table 3), and cluster and multidimensional analyses (Figures 3 and 4). According to the matrix of M value (Table 3) the population of Arsanjan had the lowest migration rate to the other test populations. Small relationships between this population and others can also be deduced by comparing the geographic (Figure 1) and geometric maps according genetic distances (Figure 4). In Figure 4 FA is farther from other populations but this is not true in the geographic map. Arsanjan is in a warm semi-desert region surrounded by mountains that separate it from other test populations. In addition, collection sites in Arsanjan were old and partly abandoned orchards without any suitable pest control treatment and probably less young tree exchanges by farmers. This situation suggests the low probability of gene flow to other test populations that could affect the genetic structure of the population.

The geographic distance between Saveh (MA) and Sabzevar (KhA) is rather large (Figure 1) but the analyses showed the shortest genetic distance between them (Table 2). The Mantel plot (Figure 2) shows this distance correspond with rather low genetic and high geographic distance, and these populations have the highest migration rate (Table 3) and the most similarity with each other in cluster analysis (Figure 3). The geometric map according to genetic distance (Figure 4) shows decreasing distance between KhA and MA. MA moved from northwest in real geographic distance to northeast in genetic distance. Hence the two mentioned populations are similar to each other in spite of long geographic distance. Human transportation can play a noticeable role in creating similarity in different populations. Failloux et al. 1997 found significant correlation between gene flow among populations of *Aedes polynesiensis* and human transportation instead of geographic distances. There are some other similar examples of allopatric populations of other insects (Julvez et al. 1990; Julvez and Mouchet 1994; Chen et al. 2004; Goodisman et al. 2001). Transportation of pomegranate fruit and young trees is presently being done by the private sector in Iran that makes it impossible to trace movement of them. However Saveh is known as one of the most famous centers of some popular varieties of pomegranate in Iran, hence, transporting pomegranate fruits and young trees to other places seems to be very possible. The short genetic distance and the highest gene flow (Table 3) between Saveh (MA) and Sabzevar (KhA) suggest high transportation between these places.

Comparisons of *E. ceratoniae* populations on different hosts were performed to detect any host fidelity in test populations. Analysis of molecular data did not show any significant difference between putative host-associated populations in the same locations and non-significant FIT (*p*>0.001) in AMOVA shows variation in average heterozygotes in populations that are probably the result of many random matings between sympatric and quasi-sympatric populations on different hosts. Downie et al. 2001 examined the distribution of molecular variation in an aphid (*Daktulosphaira vitifoliae*) and according to their results, differentiation was found to be only in allopatric or parapatric populations but no evidence was found for such differentiation in the two hosts in sympatry.

According to the results of our study, *E. ceratoniae*, which emerges in late April in Iran lays eggs on any available suitable host. Apparently the special condition of the neck in pomegranate fruit provides an optimum site for oviposition. If other hosts provide suitable conditions such as the tracks and grooves in pistachio (Mehrnejad 2002), adult moths can also lay their eggs on them. Observations during specimen collection showed low levels of pest infestation on hosts other than pomegranate by *E. ceratoniae.* Larvae of *E. ceratoniae* that grow in pistachio, fig and walnut would become larger moths than those on pomegranate (Mozaffarian et al. 2007), hence the ability for expanding host range in second half of the growth season can result in more successful over-wintering and an increase in population size. High variation among individuals (85.51% of total variation) is evidence of different genotypes in the evaluated populations. It is also supported by significant Fis (*p*= 0.00000+-0.00000) indicating significant variation among individuals of all populations.

Since the individuals of any test population feed on the same host and in the same locality, the observed variation may lead populations to adapt and survive in difficult conditions such as the stress caused by control practices. Sluss and Graham (1979) showed high levels of intra population variation and gene flow among populations of *Heliothis virescens*, as was the case in *E. ceratoniae* in our study. They concluded that populations may rapidly respond to control measures and subsequent resistance would be spread throughout the continental populations, as shown in natural populations. Fang et al. 2005 found high level of polymorphism within each population of two thrips (*Thrips tabaci* and *Frankliniella occidentalis*) and low polymorphism among populations that showed high level of heterozigosity and significant level of sexual reproduction for both species. In contrast, Benavides et al. 2005 found low genetic variability along with high differentiation among coffee berry borer populations and they suggested that the pest may lack the genetic variability necessary to respond to an intensive control strategy.

It seems that there is enough genetic variation in natural populations of *E. ceratoniae* to show alternative phenotypes in response to control practices such as omitting places for over-wintering or sites for oviposition. Infecting other fruits, hatching on the other parts of the pomegranate fruit instead of neck such as skin and cracks of the fruit have been recorded in low density (Shakeri 2004), and are alternatives that may be selected against control practices. The ability of *E. ceratoniae* to infect citrus that was observed in the laboratory (unpublished data), and some unconfirmed records of the presence of larvae in citrus in northern Iran, suggests broadening of the host range of this pest in the future. These adaptations can easily spread to other populations *via* the high level of gene flow.

## Abbreviations

AFLP: Amplified fragment length polymorphism;
Po: Pomegranate;
Pi: Pistachio;
Fi: Fig;
Wa: Walnut
